# Biparametric prostate MRI: impact of a deep learning-based software and of quantitative ADC values on the inter-reader agreement of experienced and inexperienced readers

**DOI:** 10.1007/s11547-022-01555-9

**Published:** 2022-09-17

**Authors:** Stefano Cipollari, Martina Pecoraro, Alì Forookhi, Ludovica Laschena, Marco Bicchetti, Emanuele Messina, Sara Lucciola, Carlo Catalano, Valeria Panebianco

**Affiliations:** Department of Radiological Sciences, Oncology and Pathology, Sapienza University/Policlinico Umberto I, Viale Regina Elena 324, 00161 Rome, Italy

**Keywords:** Multiparametric MRI, Biparametric MRI, Prostate cancer, Quantitative ADC, Artificial intelligence, Inter-reader agreement

## Abstract

**Objective:**

To investigate the impact of an artificial intelligence (AI) software and quantitative ADC (qADC) on the inter-reader agreement, diagnostic performance, and reporting times of prostate biparametric MRI (bpMRI) for experienced and inexperienced readers.

**Materials and methods:**

A total of 170 multiparametric MRI (mpMRI) of patients with suspicion of prostate cancer (PCa) were retrospectively reviewed by one experienced and one inexperienced reader three times, following a wash-out period. First, only the bpMRI sequences, including T2-weighted imaging (T2WI), diffusion-weighted imaging (DWI) sequences, and apparent diffusion coefficient (ADC) maps, were used. Then, bpMRI and quantitative ADC values were used. Lastly, bpMRI and the AI software were used. Inter-reader agreement between the two readers and between each reader and the mpMRI original reports was calculated. Detection rates and reporting times were calculated for each group.

**Results:**

Inter-reader agreement with respect to mpMRI was moderate for bpMRI, Quantib, and qADC for both the inexperienced (weighted k of 0.42, 0.45, and 0.41, respectively) and the experienced radiologists (weighted k of 0.44, 0.46, and 0.42, respectively). Detection rate of PCa was similar between the inexperienced (0.24, 0.26, and 0.23) and the experienced reader (0.26, 0.27 and 0.27), for bpMRI, Quantib, and qADC, respectively. Reporting times were lower for Quantib (8.23, 7.11, and 9.87 min for the inexperienced reader and 5.62, 5.07, and 6.21 min for the experienced reader, for bpMRI, Quantib, and qADC, respectively).

**Conclusions:**

AI and qADC did not have a significant impact on the diagnostic performance of both readers. The use of Quantib was associated with lower reporting times.

## Introduction

Prostate multiparametric MRI (mpMRI) is the most accurate imaging study for prostate cancer (PCa) diagnosis, and it is increasingly used worldwide for early detection, staging, follow-up, and active surveillance [1–3]. The PI-RADS recommendations, describing a standardized protocol and reporting system [4], have contributed to the very high diagnostic performance in PCa detection reported by several level 1 evidence trials and a Cochrane systematic review [5–9]. An alternative to mpMRI is an abbreviated protocol known as biparametric prostate MRI (bpMRI), which combines T2-weighted imaging (T2WI) and diffusion-weighted imaging (DWI)/apparent diffusion coefficient (ADC) without using contrast media [8, 10–12]. bpMRI has the advantages of avoiding the costs and potential side effects related to the use of contrast media, and of shortening the acquisition times. It has been shown that bpMRI has a high accuracy when interpreted by experienced radiologists, whereas the available evidence suggests lower performance for less experienced readers [13–16]. Furthermore, the use of a shorter bpMRI protocol only partly addresses the problems related to the heavy clinical workload for the genitourinary radiologist. With the growing demand for prostate MRI, it is imperative to maximize the diagnostic potential of bpMRI and at the same time to optimize reporting times for each patient [17–19].

Several possible approaches may be investigated to improve the diagnostic potential of bpMRI for less experienced radiologists. An example could involve the use of quantitative data extracted from the ADC map as a decision support system for the interpretation of equivocal lesions. The PI-RADS guidelines state that diffusion-weighted imaging is the dominant sequence for scoring lesions in the peripheral zone of the prostate, which is the most common site where PCa lesions arise. The calculation of quantitative ADC (qADC) values for a particular lesion could improve the diagnostic confidence of the radiologist in scoring suspicious lesions [20–25]. This is especially the case in bpMRI where the absence of dynamic contrast enhancement (DCE) could make equivocal cases much more difficult to interpret [26].

Another potential approach to enhance the diagnostic performance of bpMRI could be the use of artificial intelligence (AI) as a computer-aided diagnosis (CAD) system. A CAD system can serve as a complete automated diagnosis tool, or as a support tool for the radiologist with the goal of improving diagnostic accuracy and/or productivity [27, 28]. In recent years, numerous machine learning (ML) and deep learning (DL) algorithms have been applied to medical imaging and prostate MRI, mostly in preliminary research settings [29–31]. AI algorithms can be adapted to a variety of different tasks in prostate imaging, including quality control, segmentation, detection, and characterization [27].

The aim of this study was to evaluate the use of qADC measurements and an AI-based CE-approved software (Quantib Prostate) in the interpretation of prostate bpMRI, with focus on inter-reader agreement, performance in detecting PCa, and reporting time.

## Material and methods

### Patient population and MRI protocol

The study retrospectively included mpMRI studies performed at our institution for suspicion of PCa during the period of May 2021 through November 2021, with waiver of informed consent approved by the institutional review board. Inclusion criteria were the availability of all three mpMRI sequences (T2WI, DWI/ADC, DCE) and of the official PI-RADS v2.1-compliant mpMRI report provided by a senior genitourinary radiologist (VP) with 15 years of experience at a high-volume referral center in prostate diagnostics (> 1000 prostate mpMRI studies read per year). Exclusion criteria were represented by inadequate image quality of one of the bpMRI sequences (T2WI, DWI or ADC), assessed according to PI-QUAL parameters [32].

MRI examinations were performed on a 3.0 Tesla MRI (GE Discovery 750, GE Healthcare, Milwaukee, USA), using a 32-channel surface phased-array body coil (TORSOPA), with a PI-RADS v2.1-compliant protocol, consisting of a high-resolution T2WI in axial and coronal planes. During DWI, b values were set at 50, 800, and 1500; the ADC map was computed with b values of 50 and 800. Perfusion imaging (DCE) was performed following intravenously by gadobutrol (0.1 mmol/kg). Table 1 contains a detailed list of MRI protocol parameters. The patients were instructed to perform a rectal enema 2–4 h before the test.

**Table 1:** Multiparametric MRI acquisition parameters

	T2WI	DWI	DCE
Sequence type	Fast Recovery Fast Spin Echo (FRFSE)	Echo Planar Imaging (EPI)	LAVA Gradient-Echo
Acquisition plane	Axial & Coronal	Axial	Axial
Number of averages	6	2 (b 50); 6 (b 800); 12 (b 1500)	1
Slice thickness (mm)	3	3	4
Matrix size	320 × 224	90 × 90	160 × 140
Field of View (cm)	18 × 18	20 × 20	18 × 18
b-values	N/A	50–800-1500	N/A
Temporal resolution (s)	N/A	N/A	6
Contrast media	N/A	N/A	Gadobutrol 0.1 mmol/Kg

### Image interpretation and analysis

**Fig. 1:**
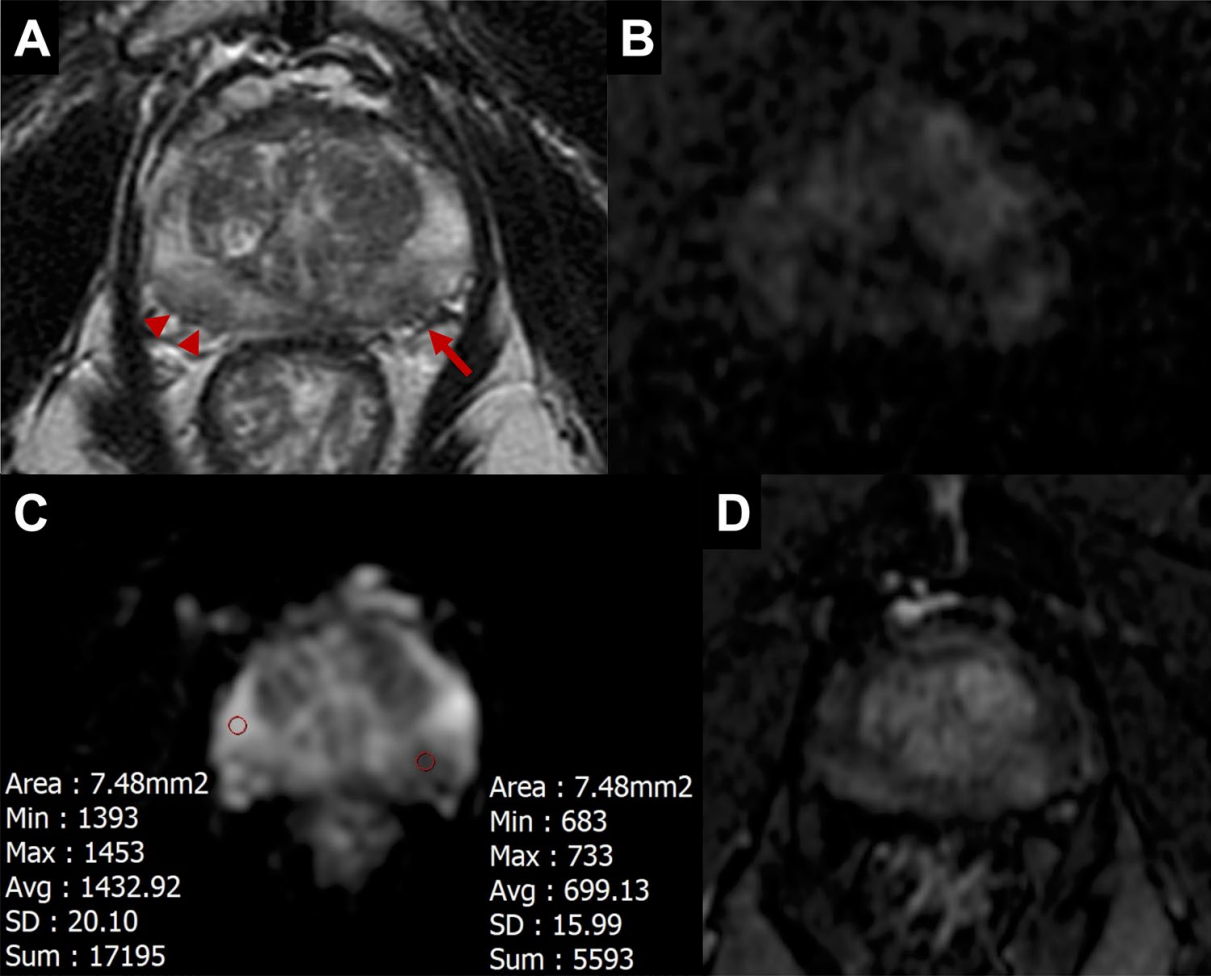
Sixty-three-year-old biopsy-naïve male with a serum PSA of 5.03 ng/ml and a PSA density of 0.15. A. T2WI showing hypointense foci in the left (arrow) and right (arrowheads) postero-lateral peripheral zone, more apparent on the left. B. DWI at b value of 1500 showing moderate hyperintensity of the two foci, more evident on the left. C. ADC map showing marked hypointensity of the left postero-lateral focus; quantitative ADC measurements reveal an ADC ratio of 0.49. D. Early phase DCE image revealing enhancement of both foci mpMRI assigned a PI-RADS score of 4 to both foci, based on the positive DCE. On bpMRI with qADC, a PI-RADS score of 4 was assigned to the left focus based on an ADC ratio < 0.59, and a PI-RADS score of 3 was assigned to the right focus, based on an ADC ratio > 0.59. Histopathology showed a Gleason 3 + 4 PCa on the left side focus and inflammatory changes on the right-side focus.

MRI studies were independently interpreted by one inexperienced and one experienced radiologist (AF, SC). The inexperienced radiologist had 3 months of experience in prostate imaging, had received fellowship-level training comprising theoretical lectures and practical training including supervised reading of approximately 100 cases, and had independently read 50 mpMRI studies before the beginning of the study. The experienced reader had 5 years of experience in prostate imaging, had completed fellowship training, and had read > 1000 total mpMRI studies (> 200 per year). Both readers, blinded to the mpMRI results, interpreted the bpMRI studies and scored any identified lesions according to PI-RADS v2.1 recommendations. Given that only the T2WI and DWI/ADC sequences were evaluated, lesions in the peripheral zone were scored as if the DCE was negative. Lesion number, location, and PI-RADS score were recorded. Each radiologist interpreted the MRIs three separate times, using a wash-out period of at least three weeks between each reading for memory extinction. The first reading was based on interpretation of the bpMRI sequences (T2WI, DWI, ADC). The second reading was performed using the bpMRI sequences, as well as the normalized ADC value for the suspicious foci to provide additional insight into the suspicion level of each focus. For the calculation of qADC, a circular region of interest (ROI) was placed independently by each radiologist interpreting the study on each suspicious focus in the ADC map at the area corresponding to the highest signal intensity at high b values in DWI. An equally sized ROI was placed on the normal-appearing peripheral zone or transition zone (according to the location of the suspicious focus) on the same slice as the lesion to normalize the qADC value (Fig. 1). The average pixel value of the lesion ROI was divided by the average pixel value of the normal prostate ROI to yield an ADC ratio. As an additional parameter for scoring suspicious lesions, the resulting ratio was considered as significant for upgrading PI-RADS 3 lesions to PI-RADS 4, using a threshold of 0.59 [26]. The third reading was performed using the Quantib Prostate v1.2.0 software, using the bpMRI sequences, as detailed below. Reporting times were recorded for both radiologists.

### Quantib prostate work-up

**Fig. 2:**
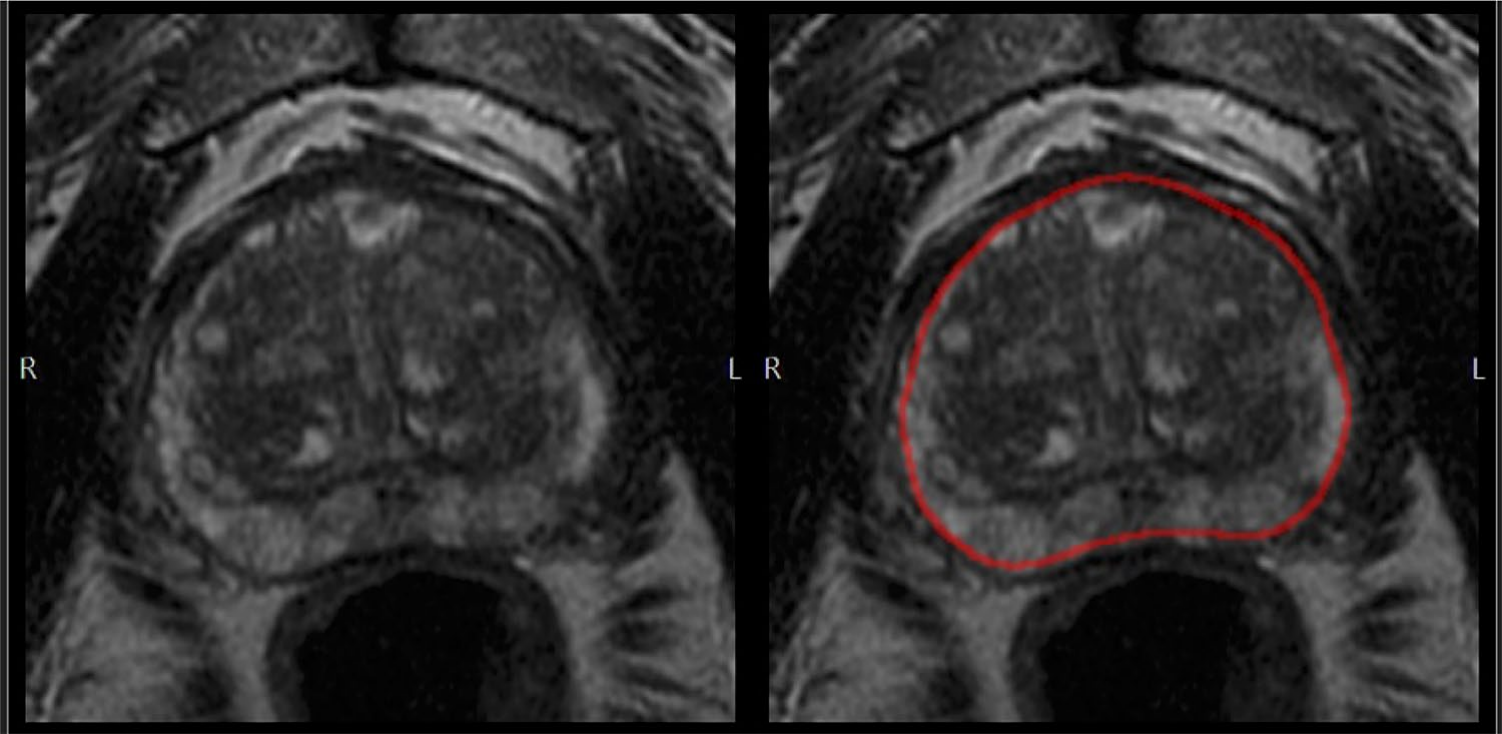
Automatically generated prostate contour overlaid on the axial T2WI is the first step of the Quantib Prostate workflow. The user can manually modify the segmentation, if needed, before approving it

**Fig. 3:**
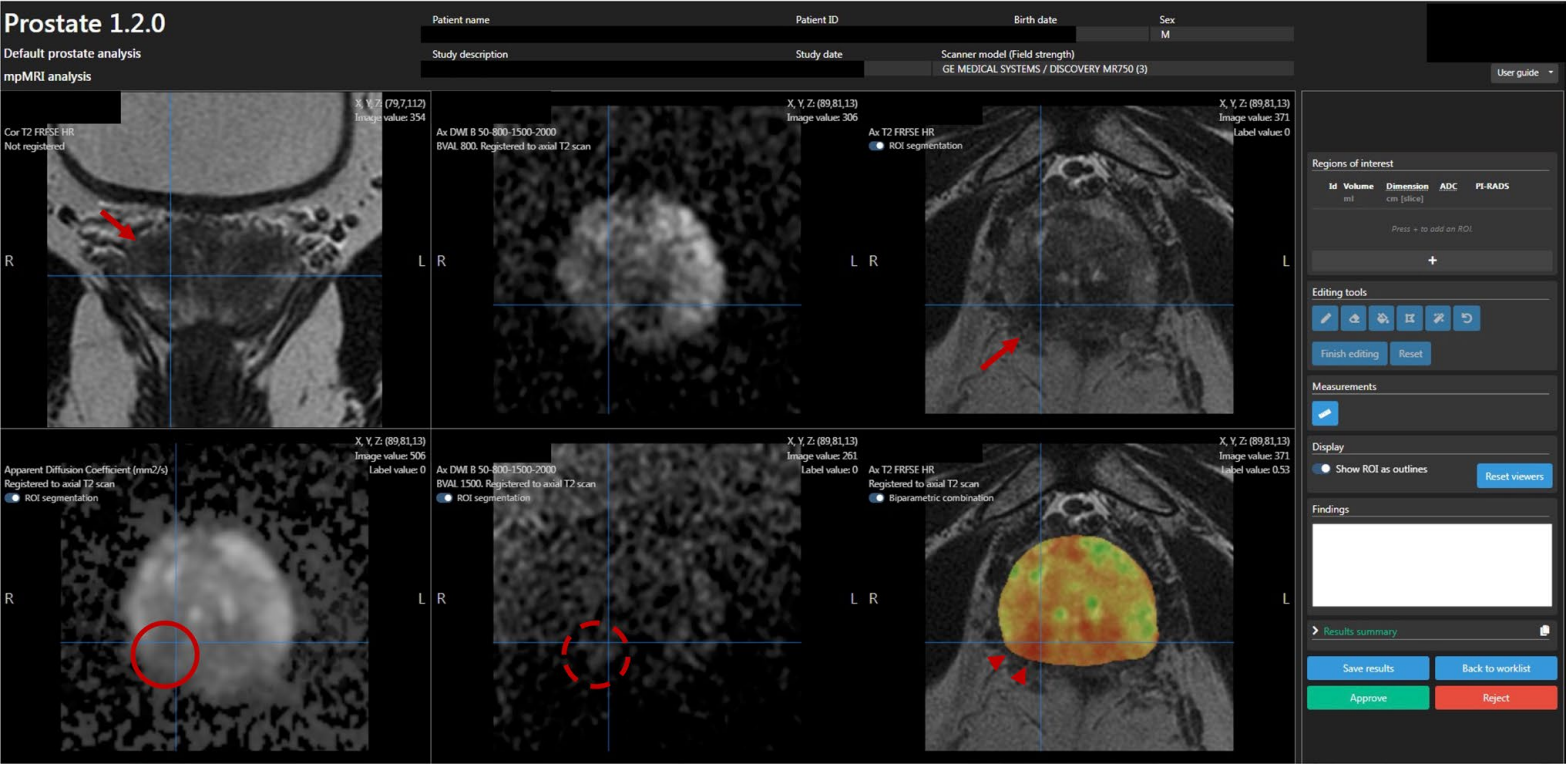
Seventy-five-year-old biopsy-naïve male with a serum PSA of 12.7 ng/ml and a PSA density of 0.48. Quantib Prostate interface for the viewing/reporting step. The bpMRI sequences are shown, in order: coronal T2WI, DWI at b value of 800, axial T2WI, ADC map, DWI at b value of 1500, colorimetric map overlaid on the axial T2WI. bpMRI images show a hypointense focus on the T2WI, in the right postero-lateral peripheral zone at the prostate base (arrows). The lesion doesn’t show hyperintensity on the high b value DWI (dashed circle). The ADC map shows only moderate hypointensity (solid circle). A PI-RADS score of 3 would have been assigned to the lesion based on the bpMRI sequences. The AI-generated colorimetric map on the bottom right shows a markedly “hot” (red) area corresponding to the right postero-lateral focus (arrowheads), suggesting a high probability focus; the final PI-RADS score assigned was 4, based on the colorimetric map. Histopathology revealed a Gleason score 3 + 4 PCa

Quantib® Prostate (Quantib BV, Rotterdam, The Netherlands) is an FDA and CE-approved MRI viewing and reporting platform based on deep learning (DL). During the Quantib Prostate work-up, the first step was the semi-automated generation of a segmentation contour of the prostate gland based on the T2WI axial sequence (Fig. 2), which was reviewed by the radiologists and manually edited, if necessary. The second step was image interpretation on the DICOM viewing interface of the software that showed both the bpMRI sequences and an automatically generated colorimetric map based on Convolutional Neural Networks (CNNs), overlaid on T2WI images, that shows in different colors the voxels that are more likely to have clinically significant PCa (csPCA, ISUP Grade ≥ 2) (Fig. 3). In this step, the radiologists were able to identify and score suspicious lesions by directly clicking on them on the MRI images. In the last step, the final report was automatically generated, manually edited if required, and exported by the software.

### Statistical analysis

The inter-reader agreement on lesion score was determined between the two readers for all three interpretation methods as well as between each reader and the mpMRI findings using the weighted Cohen’s kappa. In addition, a Cohen's kappa score was also calculated at both patient and lesion levels for presence or absence of suspicious foci (PI-RADS ≤ 2 vs. PI-RADS ≥ 3). Agreement was considered slight for kappa values of 0.00–0.20, fair for values of 0.21–0.40, moderate for values of 0.41–0.60, substantial for values of 0.61–0.80, almost perfect for values of 0.81–1.00. Detection rate was defined as the ratio of detected PCa cases to the total number of cases, using histopathology results of MRI-TRUS fusion targeted biopsy as the reference standard. Detection rate was calculated both in terms of csPCa detection (ISUP Grade ≥ 2) and overall PCa detection for the three interpretation methods (bpMRI, Quantib, qADC) and for mpMRI. One-way ANOVA was used to compare reporting times among the three patient groups for both the inexperienced and experienced readers. Statistical significance was defined as a p value less than 0.05. All statistical analyses were performed using the R statistical software version 4.1.0 (R Foundation for Statistical Computing, Vienna, Austria).

## Results

A total of 204 mpMRI studies were evaluated for inclusion in the study. Thirty-four MRI examinations were excluded due to inadequate quality on at least one of the bpMRI sequences. The 170 patients included in the study were homogeneous between the bpMRI, Quantib, and qADC groups with regard to median age (65 vs. 68 vs. 64 years old, p = 0.10), median total PSA value (7.6 ng/ml vs. 7.9 ng/ml vs. 7.3 ng/ml, *p* = 0.72), and median PSA density (0.13 ng/ml/ml vs. 0.11 ng/ml/ml vs. 0.13 ng/ml/ml, *p* = 0.64).

At mpMRI, a total of 238 lesions were identified by the senior radiologist (median of 1 lesion per patient), of which 64 (26.9%) PI-RADS 2 lesions, 73 (30.7%) PI-RADS 3 lesions, 90 (37.8%) PI-RADS 4 lesions, and 12 (5.0%) PI-RADS 5 lesions. Overall, 128/170 patients (75.3%) had at least one lesion classified as PI-RADS 3 or greater. The three study groups had a similar distribution with regards to the PI-RADS scoring of the lesions (*p* = 0.99, Table 2). One hundred and two patients underwent MRI-TRUS fusion targeted biopsy, of which 35/102 (34.3%) patients had negative results, 17/102 (16.7%) patients had Gleason Score 3 + 3 PCa, and 50/102 (49.0%) patients had Gleason Score ≥ 3 + 4 PCa. There were no statistically significant differences among the study groups in the proportion of overall PCa and clinically significant PCa (*p* = 0.91, Table 2).

**Table 2:** Patient characteristics

Variable	bpMRI (*n* = 56)	Quantib (*n* = 57)	qADC (*n* = 57)	*p* Value
Age, years *Median (IQR)*	65 (60–71)	68 (60–71)	64 (62–75)	0.10
PSA, ng/ml *Median (IQR)*	7.6 (4.52 – 8.56)	7.9 (5.02 – 8.87)	7.3 (4.63–9.01)	0.72
PSA density, ng/mL/cc *Median (IQR)*	0.13 (0.09 – 0.16)	0.11 (0.08 – 0.18)	0.11 (0.10–0.19)	0.64
PI-RADS v2, n (%)*				
*2*	24 (26.1)	25 (26.6)	27 (28.7)	0.99
*3*	30 (32.6)	29 (30.9)	28 (29.8)	
*4*	34 (37.0)	35 (37.2)	33 (31.9)	
*5*	4 (4.3)	5 (5.3)	6 (6.4)	
*Histopathology (MRI-TRUS fusion targeted biopsy), n (%)*				
Negative	11 (33.3)	13 (37.1)	15 (44.1)	0.91
*ciPCa*	6 (18.2)	7 (20.0)	5 (14.7)	
*csPCa*	16 (48.5)	15 (42.9)	14 (41.2)	
Variable	bpMRI (n = 56)	Quantib (n = 57)	qADC (n = 57)	*p* Value
Age, years *Median (IQR)*	65 (60–71)	68 (60–71)	64 (62–75)	0.10
PSA, ng/ml *Median (IQR)*	7.6 (4.52 – 8.56)	7.9 (5.02 – 8.87)	7.3 (4.63–9.01)	0.72
PSA density, ng/mL/cc *Median (IQR)*	0.13 (0.09 – 0.16)	0.11 (0.08 – 0.18)	0.11 (0.10–0.19)	0.64
PI-RADS v2, n (%)*				
*2*	24 (26.1)	25 (26.6)	27 (28.7)	0.99
*3*	30 (32.6)	29 (30.9)	28 (29.8)	
*4*	34 (37.0)	35 (37.2)	33 (31.9)	
*5*	4 (4.3)	5 (5.3)	6 (6.4)	
*Histopathology (MRI-TRUS fusion targeted biopsy), n (%)*				
Negative	11 (33.3)	13 (37.1)	15 (44.1)	0.91
*ciPCa*	6 (18.2)	7 (20.0)	5 (14.7)	
*csPCa*	16 (48.5)	15 (42.9)	14 (41.2)	

^*^According to the mpMRI report

The mean qADC value of identified lesions was 0.56 (± 0.16 SD) for the inexperienced reader and 0.58 (± 0.18 SD) for the experienced reader.

The clinical, radiologic, and pathologic characteristics of the three different patient groups are summarized in Table 2.

### Inter-reader agreement between experienced and inexperienced readers

The inter-reader agreement for lesion scoring between the experienced and inexperienced readers was fair (*k* = 0.38, *p* < 0.00001) for bpMRI, moderate (*k* = 0.41, *p* < 0.00001) for Quantib and moderate (*k* = 0.41, *p* < 0.00001) for qADC.

The inter-reader agreement for lesion significance between the two readers was moderate (*k* = 0.40, *p* < 0.00001) for bpMRI, moderate (*k* = 0.41, *p* < 0.00001) for Quantib, and fair (*k* = 0.39, *p* < 0.00001) for qADC.

The inter-reader agreement for patient significance between the two readers was moderate for both bpMRI (0.42, *p* < 0.00001), Quantib (0.44, *p* < 0.00001), and qADC (0.41, *p* < 0.00001).

### Inexperienced radiologist analysis

With respect to mpMRI, the inter-reader agreement for the inexperienced radiologist was moderate at both the per-lesion analysis (*k* = 0.42, *p* < 0.00001 for bpMRI, *k* = 0.45, *p* < 0.00001 for Quantib, *k* = 0.41, *p* < 0.00001 for qADC), the significant lesion analysis (*k* = 0.44, *p* < 0.00001 for bpMRI, *k* = 0.46, *p* < 0.00001 for Quantib, *k* = 0.42, *p* < 0.00001 for qADC) and the per-patient analysis (*k* = 0.43, *p* < 0.00001 for bpMRI, *k* = 0.44, *p* < 0.00001 for Quantib, *k* = 0.42, *p* < 0.00001 for qADC), Table 3. Detection rate of PCa was 0.29 for mpMRI, 0.24 for bpMRI, 0.26 for Quantib, 0.23 for qADC. Detection rate of csPCa was 0,20 for mpMRI, 0.16 for bpMRI, 0.17 for Quantib, 0.14 for qADC (Table 4).

**Table 3:** Summary of results—inter-reader agreement between mpMRI and the three studies

	bpMRI	Quantib	qADC
*Inexperienced*			
Lesion score	0.42 (*p* < .00001)	0.45 (*p* < .00001)	0.41 (*p* < .00001)
Lesion significance	0.44 (*p* < .00001)	0.46 (*p* < .00001)	0.42 (*p* < .00001)
Patient significance	0.43 (*p* < .00001)	0.44 (*p* < .00001)	0.42 (*p* < .00001)
*Experienced*			
Lesion score	0.44 (*p* < .00001)	0.46 (*p* < .00001)	0.42 (*p* < .00001)
Lesion significance	0.43 (*p* < .00001)	0.45 (*p* < .00001)	0.42 (*p* < .00001)
Patient significance	0.45 (*p* < .00001)	0.47 (*p* < .00001)	0.43 (*p* < .00001)

**Table 4:** Summary of results—detection rate

	Overall PCa	csPCa
*Inexperienced*		
bpMRI	0.24	0.16
Quantib	0.26	0.17
qADC	0.23	0.14
*Experienced*		
bpMRI	0.26	0.18
Quantib	0.27	0.19
qADC	0.27	0.16
mpMRI	0.29	0.20

The average time needed to the inexperienced radiologist for the overall reporting of MRI examinations was 8,23 min (IQR: 5,32–10,13 min) for the bpMRI, 7,11 min (IQR: 4,43–9,36 min) for Quantib, 9,87 min (IQR: 5,72–12,01 min) for qADC. The difference between the three groups was statistically significant (*p* < 0.00001).

### Experienced radiologist analysis

The inter-reader agreement for the experienced radiologist was moderate at both the per-lesion analysis (*k* = 0.44, *p* < 0.00001 for bpMRI, *k* = 0.46, *p* < 0.00001 for Quantib, *k* = 0.42, *p* < 0.00001 for qADC), the significant lesion analysis (*k* = 0.43, *p* < 0.00001 for bpMRI, *k* = 0.45, *p* < 0.00001 for Quantib, *k* = 0.42, *p* < 0.00001 for qADC) and the per-patient analysis (k = 0.45, *p* < 0.00001 for bpMRI, *k* = 0.47, *p* < 0.00001 for Quantib, *k* = 0.43, *p* < 0.00001 for qADC), Table 3. Detection rate of PCa was 0.29 for mpMRI, 0.26 for bpMRI, 0.27 for Quantib, 0.27 for qADC. Detection rate of csPCa was 0,20 for mpMRI, 0.18 for bpMRI, 0.19 for Quantib, 0.16 for qADC (Table 4).

The average time needed to the experienced radiologist for the overall reporting of MRI examinations was 5,62 min (IQR: 3,54–9,13 min) for the bpMRI, 5,07 min (IQR: 3,43–8,76 min) for Quantib, 6,21 min (IQR: 4,32–12,01 min) for qADC. The difference between the three groups was statistically significant (*p* = 0.00001).

## Discussion

The results of this study showed that the use of the software Quantib Prostate allowed the radiologist to achieve slightly higher inter-reader agreement with mpMRI, compared to just interpreting bpMRI sequences, both in the case of experienced and inexperienced readers. The agreement between the experienced and inexperienced readers was comparable, varying slightly between fair and moderate for bpMRI, Quantib, and qADC, with a minor trend toward higher agreement with the use of Quantib. Neither Quantib Prostate nor quantitative ADC measurements could increase detection rate of PCa with reference to mpMRI, for either experienced or inexperienced readers when interpreting bpMRI studies. The agreement of bpMRI with mpMRI was moderate for both bpMRI, Quantib, and qADC, a data that are in line with the available literature. The fact that detection rate is comparable to that of mpMRI suggests that cases of disagreement did not impact detection of PCa foci.

The use of Quantib Prostate was associated with a shorter reporting time, which is potentially valuable in the clinical workflow, due to the increasing demand for prostate MRI examinations, particularly for the inexperienced user due to the longer times needed to report MRI studies.

Although this study showed comparable diagnostic accuracy, several artificial intelligence and deep learning algorithms have been developed to increase or automate the interpretation of prostate MRIs [33–35]. Due to lack of extensive real-world validation, however, there are no definitive data mandating their use in clinical practice to date [36]. Despite interesting results from earliest reports of AI implementations focusing on automated detection and characterization of PCa, currently there is increasing interest for using AI technology to improve quality control and workflow efficiency in radiology [37].

The decrease in reporting times with the use of Quantib Prostate can be attributed to several factors. First, the automated segmentation allows for automated accurate calculation of the PSA density, a task that would otherwise require the radiologist to take three orthogonal measurements of the prostate gland, calculate the prostate volume, and therefore the PSA density. Second, the colorimetric map could allow for easier/quicker identification of suspicious foci, probably more relevant for the inexperienced reader. Third, once the lesions are identified, the user can use a dedicated tool and click on the lesion to start an automated segmentation of the lesion. After assigning a location and a PI-RADS score to the lesion(s), a structured report is automatically generated and exported. Furthermore, the colorimetric map calculated by the software could reveal suspicious foci that were not initially evident to the radiology on the bpMRI sequences, as it was noticed to happen occasionally in this study.

The AI software, however, was found to be sensitive to the quality of the overall image and the presence of artifacts in our dataset. Optimal image quality and typical prostate shape are needed for successful prostate segmentation algorithms. Well-defined margins are also necessary for accurate results. A user can manually correct a segmented contour if the segmentation is incorrect, but this will add to the reporting time. Further, the image analysis algorithm also requires adequate image quality to produce a colorimetric map that is accurate and contrasted sufficiently to show suspicious foci.

There are several limitations to this study, including the retrospective single-center nature of this study. Secondly, the PI-RADS scoring system was used to classify lesions using bpMRI, even though the score was originally designed to make full use of the entire mpMRI protocol. Therefore, a full agreement between bpMRI and mpMRI may not be feasible in all cases. Furthermore, the diagnostic performance was assessed in terms of detection rate for those lesions that underwent targeted biopsy. Consequently, it is not possible to estimate the true diagnostic accuracy of all readers, since we may have missed lesions at mpMRI that never underwent targeted biopsy. A true assessment of the diagnostic accuracy would require all patients to have a histology report and/or a relatively long period of clinical and radiological follow-up. In addition, it is highly probable that the performance of the inexperienced reader likely improved during the data collection stage; therefore, the calculated inter-reader agreement and detection rate represent an average representative value along the radiologist’s learning curve.

Lastly, all prostate MRI scans used in the study were obtained from a high-volume referral center with extensive experience in prostate MRI and an optimized acquisition protocol. Considering that the AI-based algorithms are quite sensitive to artifacts and degradation of image quality, the findings of this study might not be generalized to different clinical settings.

Future studies should determine whether Quantib Prostate can facilitate a faster learning curve for radiologists with limited experience in genitourinary radiology. In addition, the impact of the use of Quantib Prostate could be investigated for mpMRI in upcoming studies.

## Conclusions

In conclusion, in both experienced and inexperienced readers, the deep learning-based software Quantib Prostate was associated with slightly higher inter-reader agreement with mpMRI. Both the use of Quantib and quantitative ADC achieved similar diagnostic performance in terms of detection rate compared to using only bpMRI sequences. When using Quantib Prostate, both experienced and inexperienced readers could report bpMRI scans in a shorter amount of time.
